# Host Defence RNases as Antiviral Agents against Enveloped Single Stranded RNA Viruses

**DOI:** 10.1080/21505594.2021.1871823

**Published:** 2021-03-04

**Authors:** Jiarui Li, Ester Boix

**Affiliations:** Dpt. Of Biochemistry and Molecular Biology, Faculty of Biosciences, Universitat Autònoma De Barcelona, Spain

**Keywords:** Enveloped single-stranded RNA Viruses, virus-host interplay, RNases, innate Immunity, antiviral drugs

## Abstract

Owing to the recent outbreak of Coronavirus Disease of 2019 (COVID-19), it is urgent to develop effective and safe drugs to treat the present pandemic and prevent other viral infections that might come in the future. Proteins from our own innate immune system can serve as ideal sources of novel drug candidates thanks to their safety and immune regulation versatility. Some host defense RNases equipped with antiviral activity have been reported over time. Here, we try to summarize the currently available information on human RNases that can target viral pathogens, with special focus on enveloped single-stranded RNA (ssRNA) viruses. Overall, host RNases can fight viruses by a combined multifaceted strategy, including the enzymatic target of the viral genome, recognition of virus unique patterns, immune modulation, control of stress granule formation, and induction of autophagy/apoptosis pathways. The review also includes a detailed description of representative enveloped ssRNA viruses and their strategies to interact with the host and evade immune recognition. For comparative purposes, we also provide an exhaustive revision of the currently approved or experimental antiviral drugs. Finally, we sum up the current perspectives of drug development to achieve successful eradication of viral infections.

## Introduction

It is almost one century since the initial isolation of individual viruses and their assignment to specific diseases[1]. During these 100 years, diseases caused by viruses have posed a huge threat to human health. Particular attention is drawn by viruses that contain RNA in their genome, which are estimated to represent about 75% of the virus-related human diseases[2]. While we are still struggling to find effective drugs or vaccines to fight against human immunodeficiency virus (HIV) or Hepatitis C virus (HCV), both of which will lead to chronic diseases, more enveloped ssRNA viruses that can cause fatal respiratory symptoms have gradually (or suddenly) emerged. According to World Health Organization (WHO), the spread of severe acute respiratory syndrome (SARS) in 2003 has led to over 700 deaths[3] and the mortality rate of the Middle East respiratory syndrome (MERS), first identified in Saudi Arabia in 2012, has already reached to 35%[4]. Most recently, the dreaded COVID-19 has spread in most countries of the world and caused more than 600,000 people's death at the end of July 2020[5]. The coronavirus pandemic has become a global public health emergency[6]. Although there are more antiviral drugs currently available than 40 years ago[7], most of them are still not effective for the treatment of viral infections caused by coronaviruses (CoVs) or Respiratory Syncytial Virus (RSV). Therefore, novel strategies are urgently needed to solve this issue.

It has been proposed that host defense Ribonucleases (RNases) can act as drug candidates, which offer an alternative way of fighting against viral infections[8]. RNases, targeting a diversity of cellular RNAs, play an important role in many biological processes such as immune modulation, angiogenesis, neurogenesis, and host defense [8–16]. Already in 1968, researchers observed that patients with tick-born encephalitis had higher RNA-catalytic activity in their blood and cerebrospinal fluids[17]. Since then, the number of RNases with reported antiviral properties has increased slowly but significantly. For example, eosinophil-derived neurotoxin (EDN/hRNase 2), one member of RNase A superfamily, displays a high activity against ssRNA viruses like HIV and RSV [18,19]. Also, the antiviral mechanisms of RNase L are nowadays studied in deep: the protein is not only involved in degrading viral RNA but is also associated with the activation of interferon (IFN) production[20]. Although there are still limited references about RNases against human CoV pathogens, it has already been demonstrated that RNase L can exert a significant effect on a murine coronavirus mouse hepatitis beta CoV strain (MHV-JHM)[21].

Taken together these positive results about the antiviral properties of human RNases, this review aims to summarize the main characteristics of enveloped ssRNA viruses that nowadays represent a threat to human health, together with the main mechanisms that RNases can exert against viral infections, with the aim to provide the basis for the development of novel and safe antiviral drugs.

## Mechanism of enveloped single-stranded RNA viral infection

**Figure 1.**
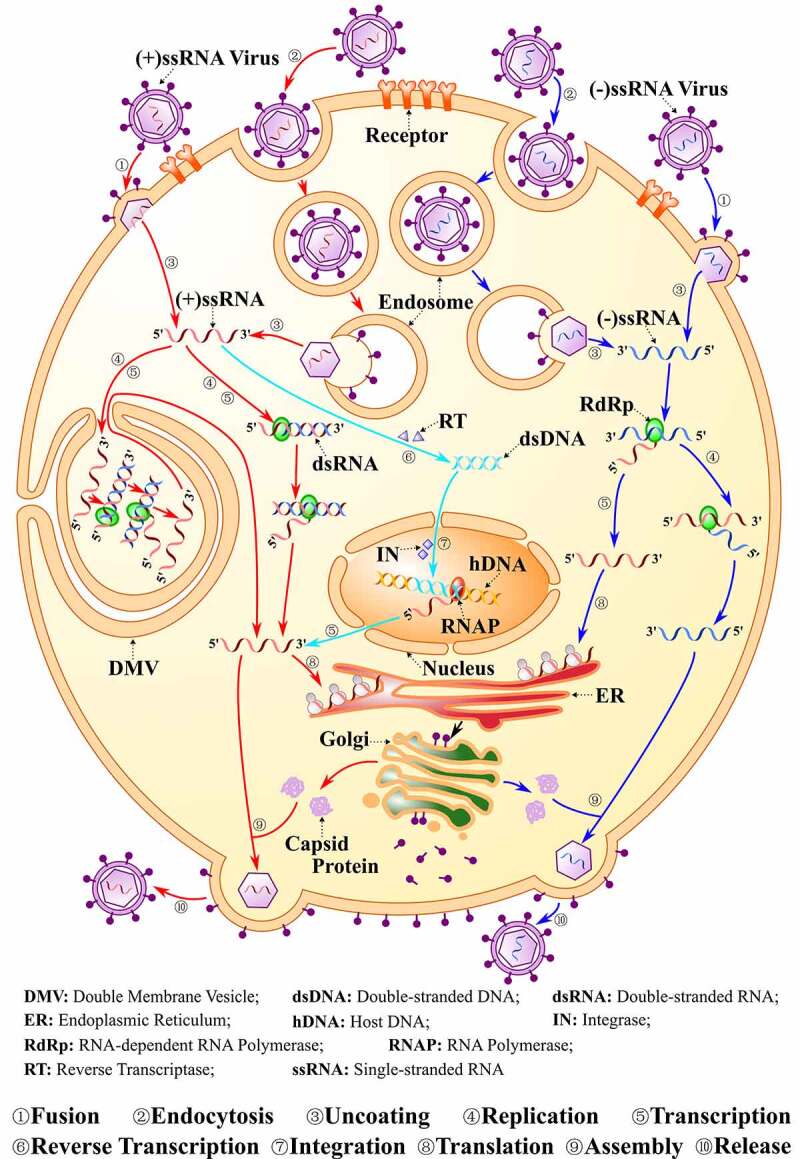
**The Viral Life Cycle**. The life cycle of positive single-strand RNA [(+)ssRNA], negative single-strand RNA viruses [(-)ssRNA] and also HIV are indicated. Normally, viruses firstly enter into the host cell by either fusion or endocytosis, then the viral genome is replicated and the viral polyproteins are translated within the cytoplasm. Many (+)ssRNA viruses can replicate and be transcribed in special DMV. Finally, virion assembly and release takes place. The life cycle for (+)ssRNA viruses are shown in red color and for (-)ssRNA in blue. For HIV, the genome will be reverse transcribed into dsDNA and then integrated into the host genome, as shown in light blue color

The entire infection viral particle, virion, consists of a nucleic acid molecule surrounded by a protein shell named capsid which, together with the genome, forms the nucleocapsid [22,23]. In particular, for enveloped viruses, there is also a lipid bilayer derived from the host cell membranes outside its nucleocapsid. Both nucleocapsid and envelope of virion contribute to the viral infection[24]. Viruses must enter into the host cells to deliver their genomic information. Fusion and endocytosis are two general ways for enveloped viruses entry (Figure 1) [25–27]. The viral membrane fusion protein can help the viruses to directly fuse its membrane with the cytoplasmic membrane [28] and fusion may also occur at the endosome after endocytosis[29]. For most enveloped viruses, the entry is mediated by a series of envelope glycoproteins such as the spike in CoVs and gp120 in HIV-1[25]. After the viral genome penetrates the host cytoplasm, the viruses will exert their function by inhibiting the normal metabolism of the host cell, activating replication of its genome, transcription and translation of its own RNA, and final assembly and release of virions[30]. Here, we will detail the structural characteristics and intracellular behaviors of some representative enveloped ssRNA viruses that recently pose a great threat to human health (Figure 1).

### Coronaviruses (CoVs) and Hepatitis C virus (HCV)

CoVs and HCV are enveloped positive ssRNA [(+)ssRNA] viruses with a diameter of about 125 nm and 40–100 nm, respectively [31,32]. The notorious viruses, SARS-CoV, MERS-CoV, and SARS-CoV-2 belong to beta-CoVs and we find eight genotypes for HCV [33,34].

The CoVs can enter into the cell either by fusion or by endocytosis[35]. The entrance into human cells relies on a receptor recognition mechanism. The spike protein (S), a glycoprotein trimer that belongs to class I fusion protein at the virion surface[36], binds to the angiotensin-converting enzyme 2 (ACE2) for SARS and SARS-CoV-2 and to the dipeptidyl peptidase 4 (DPP4) for MERS-CoV infection [37–40]. This recognition step is crucial to induce membrane fusion through the conformational transition of spike [41–43]. Also, there are many reports that describe HCV entrance into cells, mainly mediated by endocytosis and subsequent fusion into endosome by envelope glycoproteins, E1 and E2 [44–46].

Following entrance, the (+) ssRNA of the viruses can serve as both genome and mRNA and is directly translated into protein by host ribosomes[47]. The replication of the positive strand will produce a double-stranded RNA (dsRNA), which is then transcribed into a positive single-stranded genome/mRNA. Notably, the replication and transcription of (+)ssRNA viruses can take place in membrane invaginations in order to increase the efficiency of replication and evade host defense[48]. The taken membrane may be derived from various organelles, including endoplasmic reticulum (ER), late endosome/lysosome, or mitochondrial outer membrane[49]. For example, accumulation of dsRNA has been found to locate into the double-membrane vesicles (DMVs) induced by SARS-CoV infection, suggesting the probable site of viral RNA synthesis[50].

The CoV genome is the largest one among positive-strand RNA viruses. It expresses two co-terminal polyproteins, pp1a and pp1ab, which are processed into coronavirus non-structural proteins with specific biological properties, named nsp1-16 [31,51]. The endonuclease nsp15, targeting viral polyuridine sequences, participates in the evasion of dsRNA sensors. Accordingly, the loss of nsp15 activity leads to strong attenuation of the disease in mice by stimulating the immune response through activation of interferon (IFN), protein kinase R(PKR), and the 2′-5′ oligoadenylate synthetase (OAS)/RNase L system [52,53]. Likewise, the exoribonuclease, nsp14 is crucial for replication proofreading and also works as a (guanine-N7) methyltransferase (N7-MTase) for mRNA capping [54,55]. The nsp14 protein function is also key to the development of viral resistance[56]. In addition, nsp6 in the avian CoV infectious bronchitis virus (IBV) is capable of inducing autophagy, a strategy that might aim to alter the adaptive immune response and promote degradation of host immunomodulatory proteins[57]. On the other hand, HCV only encodes a single polyprotein, which is processed by the host and viral proteases to create 10 viral proteins, including Core, E1, E2, p7, NS2, NS3, NS4A, NS4B, NS5A, and NS5B. Among these, the non-structural proteins, protease NS3/NS4A, phosphoprotein NS5A, and polymerase NS5B, are the major players in viral replication and also the main targets for the design of direct-acting antiviral agents [58,59].

### Respiratory Syncytial Virus (RSV) and Parainfluenza Virus (PIV)

RSV and PIV (PIV 1–4) belong to the *Paramyxoviridae* family, which are enveloped, spherical but negative ssRNA [(-)ssRNA] viruses with a diameter of about 150 nm for RSV and 150–300 nm for PIV [60,61]. They are both the leading cause of acute lower respiratory tract infections among children younger than 5 years [62,63].

Direct fusion with the cytoplasmic membrane by a pH-independent mode has generally been considered as the main method of viral entry, although endocytosis followed by acid-independent membrane fusion is also observed for RSV [61,64–66]. There are two main envelope glycoproteins on the viral surface, the attachment protein and the fusion protein, which facilitate the attachment and cell membrane fusion for viral entry, respectively. In the case of PIV, the attachment protein is a hemagglutinin-neuraminidase [61,67]. Interestingly, in comparison with other paramyxoviruses, the RSV fusion protein alone is enough to mediate membrane fusion as well as viral infection [68] and the glycoprotein is also an unusual attachment protein that can bind to the cell surface glycosaminoglycans containing iduronic acid, like heparan sulfate and chondroitin sulfate B[69].

The replication and transcription of this kind of virus take place in the cell cytoplasm. Unlike many positive-sense RNA viruses that are translated immediately by the host cell, the genome of a negative-sense RNA virus alone is not infectious, so the viral RNA-dependent RNA polymerase (RdRp) is needed to synthesize the positive-sense RNA [60,70]. Notably, replication and transcription are controlled by the same polymerase[71]. Two proteins, large polymerase subunit L and phosphoprotein (P), contribute to the core polymerase complex. The L protein exerts its enzyme activity to participate in RNA synthesis, capping, and cap methylation while the P protein is an essential cofactor[71]. Paramyxoviruses also can produce IFN-antagonists to evade the host IFN-mediated innate immune response[72].

### Human Immunodeficiency Virus (HIV)

There are two types of HIV, HIV-1 and HIV-2, both of which are spherical, enveloped (+)ssRNA viruses. The diameter of the virion is about 120 nm for HIV-1 and 110 nm for HIV-2 [73,74]. HIV-2, which is mainly present in West Africa, has lower mortality than HIV-1[75].

Normally, both fusions with the cell membrane and endocytic pathways have been indicated for HIV entry [76–79]. The envelope glycoproteins are gp120/gp41 for HIV-1 and gp125/gp36 for HIV-2, among which the former ones (gp120 and gp125) recognize host CD4, CCR5, CXCR4 receptors, and the latter ones (gp41 and gp36) act as fusion or transmembrane proteins [74,80]. Therefore, all CD4-positive cells such as dendritic cells, T helper cells, macrophages, and astrocytes are susceptible to HIV[81].

The cytoplasm is the location where HIV transcription is activated. However, in contrast with CoVs that replicate in the cytoplasm[82], the HIV genome replicates in the nucleus. The RNA of HIV is reverse transcribed and copied into a linear dsDNA molecule that then enters into the nucleus and is integrated into the cell genome, where the virus remains in latency undetected by the immune recognition system[83]. The integration step is key for viral replication as well as for transcription and Tat (Trans-activator of transcription) is the viral-encoded transcription factor needed for the expression of viral genes [84,85]. Unfortunately, HIV can destroy the immune system and there is no protective immunity against HIV [81,86]. Also, lots of strategies are developed by HIV to regulate the cell autophagy and establish a chronic infection[87].

## Host-virus interplay

According to the characteristics of viruses introduced above, we find that interactions between the host and the virus can occur either to protect the host or to promote the viral infection. Generally, when the virion enters into the cell, it exerts multiple strategies to evade the host immune functions, including avoidance of identification by Pattern-Recognition Receptors (PRRs), inhibition of IFN signaling, and interference with host protein expression[88]. In its turn, the host responds to the virus by activation of the adaptive immunity, formation of Stress Granules (SGs), autophagy, or even apoptosis[89].

Many reviews have exhaustively discussed the overall host-virus interplay [88,90,91]. Here, we will focus on the interaction between the host and the virus with enveloped ssRNA, with a special emphasis on the type of viruses mentioned above.

### Pathogen-associated molecular patterns (PAMPs)

**Figure 2.**
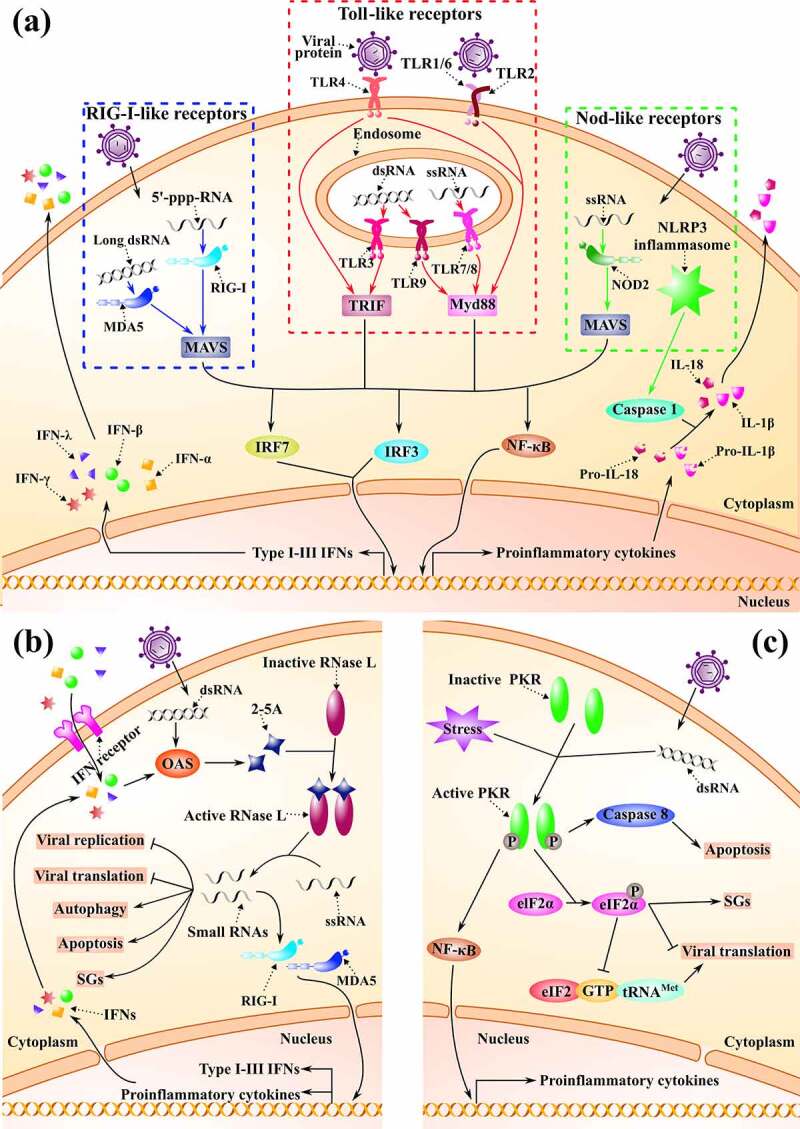
**Host-virus interplay. (a). Pathogen-associated molecular patterns (PAMPs)**. The three most common PAMPs in case of viral intrusion are shown. From left to right, they are RLRs, TLRs and NLRs respectively. Different receptors can recognize various RNAs produced by viruses. Normally, after the recognition, IFNs and cytokines are induced through a series of signal cascades. **(b). OAS/RNase L pathway**. The related processes in response to dsRNA are shown. An IFN-induced 2–5A synthetase (OAS) is expressed to synthesize 2′5′ oligoadenylates (2–5A), which activate RNase L, and then small RNAs cleaved by active RNase L can exert multiple functions to fight viruses. The related processes are shown. **(c). PKR pathway**. The important processes are indicated. PKR is also an IFN-induced, dsRNA-activated protein kinase. Once active, PKR can phosphorylate the eukaryotic translation initiation factor (eIF2α), which later suppresses viral translation and induces stress granules (SGs) formation

It is widely known that dsRNA and RNA with a 5′-triphosphate (5′-pppRNA) are commonly produced by RNA viruses during replication [92] and the host immunity will detect these “foreigners” by PRRs, such as Toll-like receptors (TLRs), RIG‐I‐like receptors (RLRs), and Nod-like receptors (NLRs) [93–95]. In TLRs, ssRNA can be identified by TLR7 and TLR8 while dsRNA by TLR3 and TLR9. Also, TLR2 and TLR4 may contribute to the recognition of viral glycoproteins[89]. In addition, TLR2 heterodimers with either TLR1 or TLR6 can provide additional recognition specificity for some viral proteins[96]. In their turn, RLRs are conformed by Retinoic Acid-Inducible gene I (RIG-I), also named DExD/H-box helicase 58, and Melanoma Differentiation-Associated protein 5 (MDA5)[97], among which RIG-I is responsible for the short RNA ligands with 5′-triphosphate caps and MDA5 for long dsRNA[92]. The recognition process of PAMPs (Figure 2a) activates IFNs signaling, as well as the expression of inflammatory chemokines and cytokines [90,98,99]. It should be noted that many key molecules such as 2′,5′-oligoadenylate synthetase (OAS), together with RNase L to form OAS/RNase L pathway (Figure 2b), and PKR (Figure 2c), are not only induced by IFN but also by dsRNA-specific PRRs that trigger the antiviral response [100,101].

Specifically, long dsRNA produced during the replication of a (+)ssRNA virus, such as the murine CoV or MHV, can be recognized by MDA5 and then activate the type I IFN response[102]. TLR3 is also a dsRNA sensor, pre-stimulation of which can block MHV infection through induction of IFN-β in macrophages[103]. Besides the recognition of dsRNA, it is demonstrated that the membrane protein from SARS-CoV can directly promote the production of IFN-β through a TLR-related signaling pathway[104]. In addition, HCV RNA can also be recognized by TLR3, MDA5, and RIG-I in infected hepatocytes and TLR7 on plasmacytoid dendritic cells (pDC), thereby inducing the secretion of type I IFN (INF-α and IFN-β) and type III IFN (IFN-λ). A recent review has detailed the interaction between the host and HCV[105].

Likewise, (-)ssRNA viruses, such as RSV, also produce dsRNA intermediates during replication. Therefore, in the RSV-infected epithelial cells, TLR3 pathways can mediate the expression of chemokines CXCL8 and CCL5[106]. Interestingly, RIG-I but not MDA5 has been identified as key for immune defense against RSV[107]. Similarly, the fusion protein of RSV contributes to the induction of interleukin 6 (IL-6), which depends on the presence of CD14 and TLR4[108]. In addition, activation of interferon-regulatory factor 3 (IRF3) and production of IFN-β was reported to be triggered by nucleotide-binding oligomerization domain 2 (Nod2), a protein that belongs to NLRs, in RSV-infected human embryonic kidney (HEK-293) cells[109].

On the other hand, the particular case of (+)ssRNA viruses, HIV, which produces dsDNA in a host cell, can also be detected by diverse PRRs. The ssRNA of HIV-1 may firstly interact with pDC, thereby stimulating INF-α production by TLR7, which later suppresses the expression of CXCR4 and CCR5 [110–112]. Concurrently, TLR8 is related to the production of IL-1β and release of Tumor Necrosis Factor α (TNF-α) by recognizing the ssRNA [113,114], whereas microRNAs produced by HIV-1 also serve as ligands for TLR8 signaling[115]. In addition, HIV-1 can be perceived by DNA sensors like Cyclic GMP–AMP synthase (cGAS) and interferon gamma inducible protein 16 (IFI16)[116]. More details about the sensing of HIV PAMPs are discussed elsewhere[117].

Unfortunately, viruses have developed many strategies to evade the host recognition. Firstly, viruses can escape from the host RNA sensors by specially modifying their RNA[118]. 2′-*O*-methylation refers to the methylation of RNA ribose at the 2′-OH position, which is common to all life kingdoms. In mammals, 2′-*O*-methylation of the 5′- guanosine cap by methyltransferases (MTases) is a molecular signature to distinguish “self” from “non-self” mRNA, a process where RIG-I and MDA5 can play a role [119–121]. Thus, 2′-*O*-methylation is one of the commonest modifications adopted by viruses to mimic eukaryotic RNA by utilizing 2′-*O*-MTases either from themselves or from the host. For example, the SARS-CoV can encode its own 2′-*O*-MTases(nsp16) and viral defective mutants are more sensitive to IFN[122]. In its turn, HIV-1 can take advantage of a cellular 2′-*O*-MTase, FTSJ3, to achieve its 5′-cap methylation[123]. In addition, N^6^-methyladenosine (m^6^A) modification is another favorite modification of viral RNA[124]. Indeed, m^6^A is the most abundant modification in mammals and regulates the mRNA stability, transport, metabolism, and efficient translation. RNA viruses are observed to incorporate this modification early in their infection cycle, in order to be properly recognized by their host machinery and facilitate translation. However, m^6^A mapping methodologies have only recently been implemented and epitranscriptomes are slowly been analyzed[125]. Therefore, the importance of this modification in viral infectivity and immune response need further exploring. Among others, this modification has been characterized during HIV-1 infection[126]. Likewise, m^6^A modification helps human metapneumovirus to evade from RIG-I [127] and may represent a key regulator of the immune system[128]. Another modification that is now recognized as a key regulator of both host and viral mRNA function is the acetylation of cytidine residues. Incorporation by HIV-1 of acetylation at N4 position (ac4C) is associated with an increase of gene expression and enhance the mRNA stability[129].

Secondly, noncoding RNAs (ncRNAs), such as transfer RNA (tRNA), ribosomal RNA (rRNA), microRNA (miRNA), small nuclear RNA (snRNA), or long non-coding RNA (lncRNA), can be derived from both the virus and the host[130]. They play important roles in host-virus interplay [131,132] and can be manipulated by the invading viruses to establish a favorable host environment for its replication cycle. Virus-host RNA interactome has been explored and a database resource has been created (ViRBase) to integrate experimental and predicted ncRNA-associated host–virus interactions [131,133]. Viral ncRNAs have a variety of biological effects that regulate the distinct steps of the viral life cycle [132,134,135]. Many examples are related to host recognition evasion: the miRNA from RSV, miR-26b, has been found to suppress TLR4 and inhibition of miR-26 can increase the expression of CCL5 and IFN-β[136]. One lncRNA, the eosinophil granule ontogeny transcript (EGOT), induced by HCV is also involved in RIG-I and PKR pathway, and inhibition of EGOT can increase the expression of IFN-stimulated genes (ISGs)[137]. On the other hand, viral mRNAs frequently incorporate Internal Ribosome Entry Sites (IRESs) sequences, which can adopt specific folds to promote their efficient translation. In addition, RNA viruses can also take profit from some host miRNAs, such as the miR-122, to facilitate their structuration and stabilize their genome or enhance the RNA translation rate. Interestingly, viruses can incorporate specific mutations in their genome to increase their stability, avoid recognition and cleavage by host proteins, or facilitate their proper folding in the absence of miRNAs helpers[138]. Besides, viruses can adopt tRNA-like structures that can intervene in their replication or translation steps [139–141]. Together with tRNA mimicry, viruses can initiate infection by taking profit from the host tRNAs. The use of the host tRNA-Lys3 by HIV-1 for priming reverse transcription was identified a long time ago, although the particular details of the tRNA priming binding site (PBS) and viral genome interactions were not elucidated till recently[142].

Besides, the viruses have also adapted strategies to evade directly the host immunity. They can produce molecules to act as antagonists against IFN induction [72,143]. We observe that most of the antagonists are non-structural or accessory viral proteins. For example, the MHV lacking nsp15 is easily detected by dsDNA sensors (such as MDA5, PKR, and OAS/RNase L), thereby stimulating more type I IFN induction. This result suggests that nsp15 may be an IFN antagonist of CoVs [52,144]. The non-structural proteins nsp1, nsp7, PLP, and the accessory proteins ORF3b, ORF6 of SARS-CoV also have been reported as potential IFN antagonists[145]. Another antagonist molecule is the RSV non-structural protein 1 (NS1), which can bind to mitochondrial antiviral signaling protein (MAVS) and suppress the association of MAVS to RIG-I and thereby undermine the IFN production[146]. Regarding HIV-1, either interaction with cellular molecules or dependence of accessory proteins, such as Vpu and Nef, can impair key signaling of PRRs including RIG-I, cGAS, and IFI16[147]. It is interesting that the core protein of HCV also can show IFN-antagonistic properties[148].

Last but not the least, positive RNA viruses can hijack intracellular membranes to form unique DMVs in order to hide their RNA and avoid the innate antiviral responses[149]. The formation of DMV owes to the nsp3-4 polyproteins for MERS-CoV and another nsp6 for SARS-CoV [150,151]. For HCV, it is indicated that several non-structural proteins as well as host factors are involved in the formation of DMVs[152]. Interestingly, in the cells infected by the negative-sense RNA virus RSV, we can find large cytoplasmic inclusion bodies that contain multifunctional proteins and even viral RNA. It is suggested that the cytoplasmic bodies may also have a protection mechanism for the replication and innate immunity evasion of negative-sense RNA viruses [153,154].

### Formation of stress granules, autophagy and apoptosis

**Stress granules** (SGs) are cytoplasmic aggregates of protein and RNA, which appear when the cell is under stress, including viral infections such as MHV and RSV [155–157]. There are two types of formation mode for SGs according to whether it depends or not on the initiation factor eIF2α, classified as type I and II[158]. Here, we briefly introduce the best-studied process, which requires the participation of eIF2α (Figure 2C). Under cell stress by a viral infection, the PKR can trigger the phosphorylation of eIF2α subunit and subsequent increase of the affinity of eIF2B for eIF2:GDP, thereby leading to the prevention of the triple complex (eIF2:GTP:tRNA^Met^) formation and shutdown of translation[159]. The SGs have either antiviral or proviral effects depending on the different studied viruses [160–162]. Antiviral SGs (avSGs) exert their specific effects by providing a platform for interaction between antiviral proteins and non-self RNA ligand, activating innate antiviral responses related to RLRs, PKR, or OAS/RNase L pathways, and IFN production. Alternatively, avSGs can inhibit the host cell translation machinery and prevent viral replication [163–166]. In their turn, some viruses manage to spread their infectivity by preventing the formation of SGs, suggesting again immune protection of SGs [164,166]. Noteworthy, we find cases where the viruses can induce SGs to serve themselves and even some possess both functions: SGs induction at early time points and SGs blockage at later stages[167]. For example, MHV-induced SGs lead to the shutdown of host translation without affecting the production of viral proteins [156] while the accessory protein 4a produced by MERS-CoV prevents the SGs formation and thereby promotes the viral replication[168]. It is also reported that HIV-1 Gag undermines both types of SGs assembly by interacting with eEF2, eIF4E translation factors, and recruitment of GTPase activating protein – SH3 domain binding protein 1 (G3BP1) [169,170]. On the other hand, the nucleocapsid of HIV-1 can induce SGs assembly, a process that can be inhibited by Staufen1, a host protein related to mRNA transport and translation [171,172]. Similarly, the role of SGs in RSV infection is still controversial[173]. The RSV-induced SGs can enhance RSV replication by mediating PKR [157] while RSV suppresses the SGs assembly by sequestering the phosphorylated p38 (p38-P) and O-linked N-acetyl glucosamine (OGN) transferase (OGT) into viral inclusion bodies[174]. In addition, RSV can also induce the specific production of tRNA-derived stress-induced RNAs (tiRNAs) and it has been found that the produced 5′-tiRNA^Glu^ inhibits the expression of antiviral protein APOER2 [175,176]. Interestingly, the authors observed that the tRNA cleavage products were mediated by the endonuclease activity of angiogenin (ANG; also named RNase 5). At the same time, it is noteworthy that ANG-induced tiRNAs are important components for host stress response and SGs assembly [177,178].

**Autophagy** is another cellular stress response pathway that can participate in the host-virus interplay. It is a process of cell recycling, by which cells can eliminate damaged or diseased components and favor healthier cells. Host cells can identify and degrade virus intruders by a process called virophagy. Without surprise, autophagy also promotes the clearance of SGs[179]. Interestingly, the autophagy mechanism has dual functions as observed for SGs. On one hand, the autophagosomes formed can transfer viral cargos to lysosomes for degradation and activate the host's innate immune. On the other hand, the viruses also take advantage of autophagy to evade the immune system, support replication, and exit the cell[180]. Many ssRNA viruses can induce, suppress, or even take advantage of autophagy by multiple mechanisms. It is reported that Nsp6 of CoVs restricts autophagosome expansion, but there are no general rules, and CoVs can also manipulate the autophagy machinery for their benefit [181–183]. For instance, autophagy may be necessary for the formation of DMVs, which will promote the efficiency of MHV replication[184]. RSV-induced or HCV-induced autophagy also contributes to the viral replication and thereby promotes infection [185,186]. Nevertheless, HIV requires autophagy for its early replication but has also developed many strategies to inhibit autophagy to avoid its clearance. Interestingly, pro- and anti-viral roles of autophagy are undergone associated with each of the cell types in the study[87].

Last, when the cell injury caused during stress exceeds the capacity of the repair mechanisms, **apoptosis** will occur to avoid excessive tissue damage. There are many reviews focused on viral infection and apoptosis[187]. For example, SARS-CoV induces apoptosis either by exposure of its membrane proteins or by its unique 7a protein [188,189] and MERS-CoV can activate both the extrinsic and intrinsic apoptosis pathways in T cells[190]. Moreover, RSV not only triggers apoptosis but can also escape or delay apoptosis by interfering in the expression of several proteins[191]. In its turn, the gp120/gp41 of HIV-1 is a key component for induction of apoptosis in CD4 T-cell lymphocytes. In contrast to other types of viruses, no evidence have been found about any mechanism of HIV to prevent apoptosis[192]. Anyhow, apoptosis is a double-edged sword. Apoptosis caused by HCV infection may seriously damage the liver while inhibition of apoptosis may lead to the persistence of HCV and subsequent development of hepatocellular carcinoma[193].

Overall, it is important to have a good understanding of the interactions that take place between viruses and their host, in order to spot the most vulnerable points during viral infection and exploit adaptive methodologies to target each type of viruses appropriately.

## Mechanism of action of host defense RNases against viral infection

Within RNases, we find endo- and exonucleases that catalyze the cleavage of either ssRNA or dsRNA and release selective cleavage products [194,195]. RNases are found in all life kingdoms; they can work either within the cell cytosol or be secreted[196]. Among the multiple biological functions of RNases, the antiviral activity has been reported [8,14,197]. Overall, the strategies exerted by RNases against viruses include: 1) inhibition of viral replication by its enzymatic activity; 2) regulation of host immune recognition and response; 3) regulation of SGs formation; 4) induction of autophagy and 5) triggering of apoptosis. Here we describe the human RNases that show significant antiviral activity together with other RNases of potential therapeutic interest. In particular, we focus on the RNases that are active against the enveloped ssRNA viruses.

### RNase A superfamily

The vertebrate-specific RNase A superfamily includes in human 13 members, named RNases 1–13, which are secreted proteins and have diverse roles, such as antimicrobial and immunomodulatory [15,18,198–201]. Expressed by innate immune cells and targeted to either the extracellular or endolysosomal space, RNase A family members are well fit to provide a safeguard action against intruding pathogens [14,202]. Within the family, we find several members with reported antiviral activity exerted by different mechanisms, depending on the type of viruses (see Table 1) [8,14,203].

**Table 1.** Anti-ssRNA viral mechanisms targeted by RNases

RNases	Proposed Mechanism (tested in vitro or in vivo)^a^	ssRNA Viruses			Refs
		Name^b^	ENV^c^	Sense	
RNase A	• Inhibits replication (in PHA-stimulated T cell blasts)	HIV-1	Y	+	^[223]^
RNase 2 /EDN	• Inhibits replication (in PHA-stimulated T cell blasts)	HIV-1	Y	+	^[204,223]^
	• Degrades viral RNA (in total RNA from infected ACH2 cells)				
	• Ribonuclease-dependent activity (in RhMk cells)	PIV	Y	-	^[19]^
	• Ribonuclease-dependent activity (in HEp-2 cells)	RSV-B	Y	-	^[19,212]^
	• A unique loop helps its interaction with viral capsid and penetration into the virion (in HEp-2 cells)				
RNase 3 /ECP	• Ribonuclease-dependent activity (in HEp-2 and THP-1 cells)	RSV-B	Y	-	^[208,209]^
RNase 5 /ANG	• Inhibits replication (in PHA-stimulated T cell blasts or PBMCs)	HIV-1	Y	+	^[220,223]^
BS-RNase	• Inhibits replication (in H9 leukaemia cells)	HIV-1	Y	+	^[224]^
Onconase	• Inhibits replication (in H9 and U937 leukaemia cells)	HIV-1	Y	+	^[224,230,231]^
	• Inhibits replication by removal of the transcription primer (tRNA synthesized *in vitro*)				
RNase L	• OAS/RNase L pathway is necessary for IFN-α but not for IFN- γ to inhibit viral replication (pancreatic islet from mouse)	CV-B4	N	+	^[268]^
	• Inhibits viral RNA synthesis (in LS1 cells)	EMCV	N	+	^[20,247,269,270]^
	• Increases the antiviral activity of IFN when overexpressed (in LS1 cells)				
	• RNase L helps to amplify IFN-β signalling (in mouse)				
	• Autophagy (in MEFs)				
	• Apoptosis				
	• UA and UU dinucleotides in viral RNA is the main cleavage site of RNase L (HCV mRNA synthesized *in vitro*)	HCV	Y	+	^[244,271]^
	• A small RNA cleaved by RNase L activates RIG-I and thereby promotes IFN-β response (in mouse)				
	• Inhibits HIV-1 replication by 8 times (in Jurkat cells)	HIV-1	Y	+	^[245]^
	• RNase L cleaves viral RNAs in absence of viral resistance protein NS1 (in A549 cells)	IAV	Y	-	^[272]^
	• IFN-γ-mediated inhibition in human epithelial cells involves the OAS/RNase L pathway (in HEp-2 cells)	RSV	Y	-	^[246]^
	• RNase L helps to amplify IFN-β signalling (in mouse)	SeV	Y	-	^[20,242,273]^
	• SGs formation (in HT1080 cells)				
	• Autophagy (in MEFs)				
	• Contributes to IFN-α/β–mediated protection (in MEFs)	SINV	Y	+	^[274,275]^
	• Interferes with the synthesis of minus strands of heat-resistant strain (in MEFs)				
	• Autophagy (in MEFs)	VSV	Y	-	^[247]^
	• Cleaves viral genomic RNA (*in vitro*)	WNV	Y	+	^[276,277]^
	• Contributes to IFN-mediated protection (in CD11b^+^ cells)				
Regnase/MCPIP1	• Inhibits replication (in hepatoma cells)	HCV	Y	+	^[278]^
	• Degrades viral RNA (*in vitro*)				
	• Degrades viral RNA (*in vitro*)	DENV	Y	+	^[263]^
		JEV	Y	+	
RNase P	• Cleaves HCV RNA transcripts (*in vitro*)	HCV	Y	+	^[264]^
Binase	• Inhibits replication (in MRC5 cells)	MERS-CoV	Y	+	^[267]^
	• Direct action on the viral mRNA (in A549 and MDCK-II cells)	IAV	Y	-	^[265,266]^

^a^A549: human lung adenocarcinoma epithelial cells; HEp-2: human laryngeal carcinoma/human type 2 epithelial cells; MDCK-II: Madin-Darby canine kidney epithelial cells; MEF: mouse embryonic fibroblasts; MRC5: human fetal lung fibroblasts; PBMC: peripheral blood mononuclear cells; PHA: phytohemagglutinin; RhMk: rhesus monkey kidney; THP-1: a human monocytic cell line. ^b^CV-B4: Coxsackie virus B4; DENV: Dengue virus; EMCV: Encephalomyocarditis virus; HCV: Hepatitis C virus; HIV: Human Immunodeficiency virus; IAV: Influenza A virus; JEV: Japanese Encephalitis virus; MERS-CoV: Middle East Respiratory Syndrome Coronavirus; PIV: Human Parainfluenza virus; RSV: Respiratory Syncytial virus; SeV: Sendai virus; SINV: Sindbis virus; VSV: Vesicular Stomatitis virus; WNV: West Nile virus. ^c^ENV: Enveloped; Y: Yes; N: No.

The Eosinophil-Derived Neurotoxin (**EDN/RNase 2**) is one of the best studied, which can fight RSV, PIV, and HIV [18,19]. Research about human recombinant EDN against RSV and PIV indicated that the ribonuclease activity of EDN is essential for the protein antiviral activity but is not unique. Indeed, bovine pancreatic RNase A, the family reference, shows a much higher catalytic activity but is devoid of antiviral activity[19]. Antiviral RNases from human chorionic gonadotropin (hCG) preparations that contain EDN show anti-HIV activity, which may be related to its inhibition of HIV replication in chronically infected ACH-2 lymphocytes and U1 monocytes[204]. The protein contribution in anti-HIV activity was proven by selective blockage with anti-EDN antibodies[205]. Besides, the addition of recombinant EDN reduces infectivity *in vitro* in RSV-infected epithelial cells [19,206]. Interestingly, EDN levels in serum are increased following RSV bronchiolitis and are used as a predictive marker of recurrent wheezing[207]. Another eosinophilic ribonuclease, the Eosinophil Cationic Protein (**ECP/RNase 3**) has also anti-RSV activity, although to a much lower extent, and no synergistic action with EDN was evidenced[208]. Very recently, we have demonstrated the antiviral activity against RSV of RNase 3 expressed in macrophages. Besides, a comparative transcriptome analysis indicates that the protein antiviral properties are associated with its catalytic activity[209]. On the other hand, increasing levels of ECP are found during HIV infection in both adults and children [210,211]. The higher antiviral activity of EDN/RNase2 with respect to ECP/RNase3 has been attributed to the presence of a specific region in the former at the C-terminal loop. By site-directed mutagenesis, the authors have identified a region essential in EDN for the RSV capsid interaction and virion entry to the cell[212].

There is no evidence that EDN/ECP has a direct effect on CoVs, but EDN is upregulated during SARS-CoV infection [213] and most recently, it has been reported that eosinopenia is associated with SARS-CoV-2 infection [214,215]. In the same line, increasing levels of eosinophils are associated with a better prognosis of recovery from COVID-19 and EDN has been proposed as a novel clinical biomarker of the disease [216–218]. Moreover, it is observed that the antiviral activity of eosinophils can be reversed by the addition of the proteinaceous RNase inhibitor (RI) [19,208].

Apart from the eosinophil-derived proteins, angiogenin (**ANG/RNase 5**) may play a role during viral infection[219]. ANG can suppress the replication of HIV-1 in a dose-dependent manner[220]. Furthermore, when infected by RSV, the cell production of tRNA halves induced by ANG is significantly enhanced. It is now known that during cell stress conditions tRNA halves promote the assembly of SGs; however, the potential contribution of ANG in the control of infection is still unclear[175]. Very recent transcriptome analysis associates epigenetic traits with the stress-induced tRNA fragment population and cell potential biological activities[221]. Interestingly, a posttranscriptional modification m^5^C, considered a protection cleavage mark in higher eukaryotes, inhibits ANG action on tRNAs[222]. Another research has also tested the anti-HIV activity of EDN, ANG, and even RNase A, all of which inhibit the HIV-1 replication during primary HIV-1-infected PHA-stimulated PBMCs[223].

Another peculiar member of the RNase A superfamily is the bovine seminal RNase (**BS-RNase**). It is the unique natural dimeric protein of the family and shows a specific enhanced activity against dsRNA. Interestingly, BS-RNase is reported to have anti-HIV activity on H9 leukemia cells[224]. In addition, dsRNA cleavage by BS-RNase is induced by IFN-γ[225]. Moreover, direct binding of the C-terminus of IFN with RNA is involved in the activation of the RNase antiviral activity[226].

Last, within the lower-order vertebrates of the RNaseA superfamily, we find amphibian RNases with an elevated antiviral activity together with other appealing potential therapeutic applications[224]. The particular attraction has been drawn by an RNase from oocytes of the leopard frog *Rana pipiens*, discovered for its antitumor properties and named **Onconase** thereafter [227,228]. Onconase has also an unusual activity on dsRNA [229] and can significantly inhibit HIV-1 replication in H9 leukemia cells at nontoxicity concentration[224]. Further work confirmed the selective degradation of the virion RNA by Onconase in treated H9 and U937 leukemia cells with no alteration of ribosomal or assayed mRNA[230]. In addition, Onconase specific activity on tRNAs has been associated with its ability to inhibit HIV replication by removal of tRNA^Lys^, which is known to serve as a reverse transcription primer by the viruses[231]. Recently, Vilanova and coworkers reported that Onconase antiviral action can also be mediated by the upregulation of the activation transcription factor 3 (ATF3) which promotes apoptosis and can inhibit the viral replication[232]. Besides, ATF3 is also reported to induce the latent state in herpes simplex virus (HSV)[233]. The authors also suggest that Onconase inhibition of HIV replication might be mediated by the induction of IL10[232]. The advantages of the clinical development of Onconase are that the protein not only avoids the inhibition by RI but is also resistant to proteolysis due to its unusual conformational stability[228]. Work is currently in progress to exploit Onconase alone or as an adjuvant to fight viral infections including MERS-CoV and RSV (http://tamirbio.com/) [232].

Overall, the induction of the apoptosis or autophagy pathways by several family members could be regarded as a strategy to facilitate the removal of pathogen-infected cells [15,234–237]. A curious and original zymogen has been engineered by Raines and coworkers, where the use of the HIV protease serves as an activation system to release the RNaseA catalytic activity that will degrade the viral genome[238].

### RNase L

RNase L is a ubiquitous intracellular endonuclease activated by a 2′5′-oligoadenylates (2–5A), which is specifically synthesized by the OAS. The OAS/RNase L pathway is activated during viral infections and is regulated by IFN[239]. Briefly, when exposed to the dsRNA produced by viruses, the host cells will induce IFN and then secrete 2–5A to induce the activation of RNase L. Activated RNase L cleaves viral ssRNA, thereby suppressing viral replication, protein synthesis, and spread. In addition, RNase L can further amplify IFN signaling and promote the formation of SGs, which include many antiviral proteins [240–242]. Thereby, RNase L provides a natural cell protection against viral infection (Figure 2b).

Many kinds of viruses such as enveloped ssRNA viruses, the object of this study, can be inhibited directly or indirectly by RNase L (Table 1) [239,243]. Activation of RNase L then generates both host and viral RNA cleavage products, which on their turn can activate RIG-I and stimulate expression of IFN-β during HCV infection [20,244]. Interestingly, HIV-1 replication can be directly suppressed by overexpression of RNase L, even without IFN treatment[245]. IFN-γ has been found to inhibit RSV infection in human epithelial cells, but cells that overexpress the RNase L inhibitor (RLI) attenuate this antiviral effect, suggesting that activated RNase L is essential for IFN-γ-mediated anti-RSV activity[246]. RNase L can also protect the brain from sustained MHV infection and thereby prevent demyelination and neurodegeneration[21]. SGs and autophagy can also be induced by RNase L in both (+)ssRNA and (-)ssRNA viral infections [242,247]. Alternatively, RNase L activation in virus-infected cells can lead to cleavage of both host and viral RNA and facilitate the activation of apoptosis and subsequent removal of infected cells[248].

Unfortunately, viruses have developed on their turn many mechanisms to evade the OAS/RNase L system. Specific 2′,5′-phosphodiesterases that cleave 2–5A are released by CoVs to prevent RNase L activation: ns4b from MERS-CoV and ns2 from MHV[249]. Deletion of the ns4b gene can activate RNase L during infection of Calu-3 lung cells and also the ns2-deletion mutant virus cannot replicate in wild-type mice but is highly pathogenic in RNase L deficient mice [250,251]. Likewise, the Tat protein of HIV-1 binds to the 2–5A synthetase, undermines the TAR-mediated activation of 2–5A synthesis, and thus blocks OAS signaling[252]. In addition, RLI can be induced during HIV-1 infection to inhibit the OAS/RNase L pathway[253]. A better understanding of the strategies of viruses to modulate the OAS/RNase L immune response pathways should provide guidance for the development of novel drugs [239,241]. In fact, avoiding the recognition of the OAS/RNase L system is not only the privilege of the popular ssRNA viruses introduced above, some other ssRNA viruses have also been found to engineer similar strategies. For example, a unique RNA structure carried by poliovirus, a (+)ssRNA virus, helps to inhibit the endonuclease activity of RNase L[254]. A specific protein (L*) produced by Theiler’s murine encephalomyelitis virus can bind to RNase L and prevent its activation by 2–5A by an antagonism stratagem similar to the one described for MHV[255]. A wealth of literature has emerged during the last months on host-virus interplay mechanisms due to COVID-19 pandemic. Latest population studies on human genetic variants related to severe COVID-19 illness have identified the deficiency in the OAS/RNase L antiviral system among the top critical phenotype markers [256,257].

### RNase T2

RNase T2 family is present not only in eukaryotes but also in bacteria and viruses, with a variety of functions, such as degradation of RNAs, regulation of the immune response, or even control of tumor progression [258,259]. RNase T2 activity has also been linked to the host antiviral response. One study demonstrated that RNase T2-deficiency resembles congenital cytomegalovirus (CMV) infection. Strikingly, CMV infection can block as an evasion strategy the antiviral RNase L response, which results in increased ssRNA levels, associated with exacerbated innate immunity activation and eventually similar neuropathological consequences[260]. This suggests that RNase T2 might play a similar role as RNase L in cellular immune response[261]. Recent research also shows that RNase T2, together with EDN, can release uridine nucleotides from pathogen RNAs, which in their turn are activators of TLR8 [202,262]. Interestingly, the enrichment in uridine products is achieved by the complementary cleavage preferences of the two endoribonucleases: RNase T2 at XU and EDN/RNase2 at UX, where X is a purine. To note, both secretory RNases work at endolysosomal compartments, with an optimum activity at a pH between 4.5 and 5.5, and might provide an indirect mechanism to activate the immune system in the presence of pathogenic RNAs. In particular, the authors have analyzed the RNases cleavage pattern on the genome of selected RNA viruses, some of which show an abundance of uridine-rich sequences.

### Regnase1 and RNase P

**MCPIP1** (also designated as **Regnase1**), is a human protein with both antiviral and endoribonuclease activities. It is a zinc finger protein involved in the cell inflammatory response that regulates the half-life of mRNA and miRNA. The nuclease domain cleaves ssRNA and shows a high affinity for viral RNA [263] and a broad-spectrum antiviral action. Likewise, another a human endonuclease, human **RNase P,** is found to cleave HCV RNA transcripts[264].

### Other RNases

**Microbial RNases** have also been proposed as therapeutic agents against ssRNA viruses (Table 1). Ilinskaya and coworkers have extensively explored the potential of the RNase from *Bacillus pumilus*, also named Binase, on influenza A virus. The authors observed a direct action on the viral mRNA in both *in vitro* and *in vivo* models [265,266]. Binase was also effectively tested against CoVs (MERS-CoV and the human low pathogenic CoV-229E strain)[267]. Other microbial RNases are extensively reviewed by Ilinskaya and Mahmud[8].

Last, a very promising emerging discipline in the field is based on the design of **artificial RNases (aRNases)** for targeted RNA cleavage. Chemical conjugates were first synthesized containing RNA binding and catalytic domains, although their main drawback was their poor catalytic efficiency[8]. Small molecule derived RNases can also be engineered by connecting short mono to tripeptides[279]. The aRNases active site includes imidazole groups and perform an equivalent acid-base catalysis to native enzymes. Although their efficiency is significantly lower than natural RNases, they are active in physiological conditions and were demonstrated effective against influenza A and H1N1 viruses. Last but not least, we can engineer antiviral **ribozymes**, RNA based-drugs with specific RNA targeting and endowed with intrinsic endonucleolytic action, some of which are in clinical trials[280].

Notwithstanding, to design effective antiviral drugs we should also consider other contributing factors, such as the three-dimensional structuration of RNA molecules, the presence of specific posttranscriptional modifications, or the involvement of RNA binding proteins that might protect the viral genome from RNases cleavage. Fortunately, novel methodologies, such as eCLIP are now providing tools to easily identify protein-RNA binding regions[281]. Some viruses can stabilize and protect their genomic RNA by interaction with the host-specific miRNAs. For example, miR122 can modify the viral RNA base pairing and increase its half-life, promoting translation and protection of HCV RNA [138,282]. The emergence of resistant virus strains to miR-122 inhibitors with enhanced genome stability and protection against host RNases has recently been reported[283]. Therefore, the ability of host RNases to target viral RNA might be hindered by the presence of a diversity of ncRNAs. To obtain a more realistic scenario of the host-virus interplay we should bring together all the intervening agents at once, RNA target molecules, RNA binding proteins, and RNA cleavage enzymes.

In summary, we can conclude that host-derived RNases can fight multiple viruses through direct or indirect actions, which not only remove viral RNAs but also regulate self-immunity. In addition, host cells have evolved inhibitors to specifically protect subcellular compartments and prevent the potential toxic action of their own RNases against cellular RNAs. Another advantage of RNases is that once expressed they can be secreted, thereby exerting their antiviral activity either at intracellular or extracellular level. This dual function is of great benefit to improve antiviral effects at the local site of infection and to reduce the potential side effects on the remaining healthy tissues. From a perspective focused on applied therapies, some nanodelivery systems have been engineered to vehicle the RNases within host cells [284–286]. We are confident that the design of novel vehicle tools should greatly expand the applicability of RNases with high catalytic efficiency on RNA viral genomes but limited cleavage specificity on target sequences.

## Current antiviral drugs design and development

It has been almost 60 years since the approval of the first antiviral drug, idoxuridine. During this period, thousands of antiviral compounds were proposed, but only about 100 antiviral drugs were approved for the treatment of major human infectious diseases[287]. The action of antiviral drugs may include inhibition of viral attachment, penetration, and uncoating, prevention of viral replication or protein synthesis, blockage of viral post-assembly and activation of innate immunity [288,289]. Among the many antiviral drugs, some can be used or even exclusively dedicated to ssRNA viral infection. Therefore, in this part, we will briefly introduce the main mechanisms for drugs that target enveloped ssRNA viral infections (summarized in Table 2). We will also take the opportunity to prospect the strategies that can be applied to develop novel drugs against ssRNA viruses.

**Table 2.** Main available drugs or candidates against enveloped ssRNA viruses

Virus		Antiviral Mechanisms	Status	Representative Drugs^d^		
(+) ssRNA virus	SARS -CoV-2	• Entry inhibitor	Investigational	• Leronlimab		
		• RNA polymerase inhibitors	Investigational /conditionally approved	• Favipiravir[323] • GS-441524	• Remdesivir	
		• Inosine-5-monophosphate dehydrogenase (IMPDH) Inhibitor	Investigational	• Merimepodib[324]		
	HCV	• NS3/4A protease inhibitors	Approved	• Asunaprevir • Boceprevir • Glecaprevir • Grazoprevir	• Paritaprevir • Simeprevir • Telaprevir • Voxilaprevir	
			Investigational	• Faldaprevir	• Vedroprevir	
		• NS5B polymerase inhibitors	Approved	• Dasabuvir	• Sofosbuvir	
			Investigational	• Beclabuvir	• Uprifosbuvir	
		• NS5A inhibitors	Approved	• Elbasvir • Daclatasvir • Ledipasvir	• Ombitasvir • Pibrentasvir • Velpatasvir	
			Investigational	• Odalasvir • Ravidasvir	Ruzasvir	
		• Interferon α/β receptor agonists	Approved	• Peginterferon α2a • Peginterferon α2b		
		• Broad-spectrum activity	Approved	• Ribavirin		
	HIV	• Nucleoside and nucleotide reverse transcriptase inhibitors (NRTI)	Approved	• Abacavir • Didanosine • Emtricitabine • Lamivudine	• Stavudine • Tenofovir disoproxil • Zalcitabine • Zidovudine	
			Investigational	• Elvucitabine	• Racivir	
		• Non-nucleoside reverse transcriptase inhibitors (NNRTI)	Approved	• Delavirdine • Doravirine • Efavirenz	• Etravirine • Nevirapine • Rilpivirine	
			Investigational	• Atevirdine • Calanolide A	• UC-781	
		• Protease inhibitors	Approved	• Amprenavir • Atazanavir • Darunavir • Fosamprenavir • Indinavir	• Lopinavir • Nelfinavir • Ritonavir • Saquinavir • Tipranavir	
			Investigational	• TMC-310911		
		• Integrase inhibitors	Approved	• Bictegravir • Dolutegravir	• Elvitegravir • Raltegravir	
		• Fusion inhibitor	Approved	• Enfuvirtide		
		• Entry inhibitors	Approved	• Maraviroc	• Ibalizumab	
			Investigational	• Cenicriviroc	• Leronlimab	
		• Others	Investigational	• BMS-488043 • Dexelvucitabine • Fiacitabine	• Lobucavir • Sorivudine	
(-) ssRNA virus	RSV	• Fusion inhibitor	Approved	• Palivizumab		
		• Broad-spectrum activity	Approved	• Ribavirin		
	Influenza virus	• CAP endonuclease inhibitor	Approved	• Baloxavir marboxil		
		• Neuraminidase inhibitors	Approved	• Oseltamivir • Peramivir		• Zanamivir
			Investigational	• Laninamivir		
		• RNA synthesis inhibitors	Approved	• Rimantadine		
			Experimental	• Triazavirin		
		• Fusion inhibitor	Investigational	• Umifenovir		

^d^Summary from DRUGBANK, category of Antiviral Agents (https://www.drugbank.ca/categories/DBCAT000066) when no other reference is provided.^341^

### Available anti-viral agents

By referring to Virus Pathogen Database and Analysis Resource (ViPR) (https://www.viprbrc.org/brc/), it is shown that more than half of approved antiviral drugs are against HIV or HCV infection, suggesting that antiviral drugs developed for human chronic viral-related diseases have received more attention and positive results. Higher percentages of approval against HIV among antiviral drugs can be confirmed in another database: http://www.virusface.com/Drug/AntiviralDrug_Compound.html. No drugs can cure HIV and HCV infections completely, but they play a key role in extending the patient’s lifespan and improving the quality of life.

Even though the concepts for the design of antiviral drugs are constantly evolving, the central rules still focus on directly targeting viruses, such as inhibitors of viral polymerases and proteases, or regulating cellular processes essential for viral replication[290]. For example, most approved anti-HIV drugs are reverse transcriptase inhibitors (RTIs) which are divided into nucleoside and nucleotide RTIs (NRTIs) and non-nucleoside RTIs (NNRTIs) [291,292]. Subsequently, protease inhibitors also account for a large proportion of antiviral agents[293]. In addition, we also find drugs acting as integrase inhibitors (Elvitegravir, Raltegravir, etc.) to block the integration of viral genetic material into human chromosomes[294], fusion inhibitor (Enfuvirtide) to prevent viral entry [295] and entry inhibitor (Maraviroc) to antagonize the interaction between HIV-1 gp120 and CCR5[296]. Similarly, most approved anti-HCV drugs target specific HCV non-structural proteins (NS), including inhibitors against NS3/4A protease, NS5B polymerase, and NS5A protein [59,297].

However, the available drugs to treat viruses that lead to severe respiratory symptoms are very limited. It is demonstrated that the broad-spectrum anti-HCV drug, Ribavirin, which blocks nucleic acid synthesis, can be used to treat RSV[298]. Besides, the approval of Palivizumab, a monoclonal antibody acting as a fusion inhibitor against RSV, opens another path for the development of alternative anti-RSV drugs based on the targeting of the F protein that mediates the virus-host fusion process. Meanwhile, other strategies are considered, such as targeting RNA polymerases or nucleocapsid mRNAs through nucleoside analogues or small-interfering RNAs (siRNAs) respectively to inhibit viral replication. However, although infants are the most vulnerable group of risk for RSV disease, most of the anti-RSV candidates that are under clinical trials have only been studied in adults [299,300].

Until now, there are no approved drugs specifically designed against CoVs, but scientists are currently committed to develop anti-CoV therapeutic agents. One representative drug under investigation is Remdesivir, a nucleoside analogue as well as a prodrug of GS-441,524 which has shown high activity against various CoVs such as SARS-CoV, MERS-CoV, MHV, and even SARS-CoV-2 either *in vitro, in vivo,* or in humans[301]. The mechanism of Remdesivir is ascribed to the inhibition of the viral RNA polymerase RdRp[302]. The nucleoside drug is also designed to block the error-proof exoribonuclease and reduce at most the capacity of viruses to acquire resistance, a property that put forward its superiority to other agents[303]. Following recent trials at the National Institutes of Health, Remdesivir, has recently obtained conditional approval for the treatment of severe COVID-19 hospitalized patients in the EU and US[304]. On the other hand, traditional antimalarial drugs, chloroquine and hydroxychloroquine, have also been proposed to treat COVID-19, with controversial results following clinical trials, uncontrolled cases, or public and media approval [305,306]. In spite of good activity against SARS-CoV *in vitro*[307], the reports of the high risk of chloroquine and hydroxychloroquine to the human cardiovascular system should not be ignored[308]. One review has summarized the treatment strategies against SARS-CoV and MERS-CoV but most are drug combination[309]. Due to the current COVID-19 pandemic, research in the field advances very rapidly and many novel strategies are waiting for final approval.

### Discovery of antiviral active compounds

According to the above discussed, we observe that most developed antiviral compounds are nucleoside or nucleotide analogues, which mimic the natural nucleosides[310]. One of the main reasons why these analogues are so effective is their ability to target viral replication. For some analogues, it is their cleavage products, released as mono-, di- or triphosphorylated nucleosides that act as the active compound[311]. For example, triphosphorylated forms of nucleoside or nucleotide analogues are competitive RTIs and nearly all NRTIs are derivatives of sugar scaffold of natural nucleosides, which will cause polymerase chain termination due to the 3′-OH modification[312]. Nucleoside (or nucleotide) analogues, including Sofosbuvir and Remdesivir, also show activity against RdRp, indicating that these analogues may work as effective inhibitors against CoV polymerases and can be candidates against COVID-19 [313,314].

Also, computational structure-based approaches have been of great help to select active compounds against viral protein or key cell receptors, which are widely used to design a new generation of antiviral drugs [315,316]. The programs can calculate the degree of interaction between a list of compounds from large databases and its target in three-dimensional models and then the top-ranked compounds are chosen for further testing *in vitro* or *in vivo* to confirm its predicted activity[317]. We find many successful examples of lead compounds in the development of anti-ssRNA viral drugs[318]. Most recently, after screening 61 molecules with antiviral activity by molecular docking studies, it was found that all tested HIV protease inhibitors, Lopinavir, Asunaprevir, Indinavir, and Ritonavir, showed significant binding interactions with COVID-19 enzymes[319]. Clinical trials also indicate that following administration of Lopinavir/Ritonavir, or both, to COVID-19 patients, lower viral loads and better clinical symptoms are achieved[320]. However, the real role of Lopinavir/Ritonavir in anti-COVID-19 treatment is still controversial[321]. Table 2 provides an overall summary of the currently available drugs, either approved or in clinical trials, to treat viral infection by ssRNA enveloped viruses.

### Current perspectives

Unfortunately, it is still difficult to find drugs that interfere with viral replication without damaging host cells. The use of nucleoside or nucleotide analogues is of a potential danger due to the risk of intake by human polymerases and incorporation into host RNA or DNA. A typical example is a mitochondrial toxicity in which nucleoside analogues interfere with mitochondrial DNA replication and thereby lead to reduced function of mitochondria[322]. It is also observed that liver, kidney, or other tissue injuries are associated with antiviral therapies by using different types of drugs [325–327]. Moreover, misfortune never comes alone; resistance to traditional antiviral drugs, either anti-HIV or anti-HCV, is continuously emerging [312,328,329]. WHO already warned in 2017 that more than 10% of the patients receiving antiretroviral therapy have a strain that is resistant to some widely used anti-HIV drugs. Similarly, HCV mutations resistance to direct antiviral drug treatments has already been well demonstrated[330].

A combination of antiviral drugs has been recommended to prevent the emergence of resistance. Complementarily, combination therapies can reduce the required amount of each drug to alleviate adverse effects. On the other hand, drugs with multiple mechanisms can target different stages of the viral life cycle. Thus, if the virus develops resistance to one drug, the others can still exert their antiviral effect. Also, fixed-dose anti-HIV combinations are available on the market that provide convenience for patients by taking one pill a day[331]. Distinct combinations have been already under investigation against viral infections to treat specific patients or to apply in case single drug treatments result is ineffective [329,332,333]. Particular interest is provided by the combination of IFN with antiviral drugs, such as Remdesivir, against CoVs[332]. The results are controversial and the treatment outcome is mostly dependent on the *in vivo* model, administration route, disease stage, or IFN type.

Another approach that is gaining popularity is the targeting of host signaling pathway rather than the virus itself to avoid toxicity and the emergence of resistance to antiviral drugs. As discussed before, an exhaustive knowledge of the interaction between the virus and the PRR signaling pathway can assist in the design of multiple antiviral therapies. Among them, we find the use of PRR agonists as adjuvants, drugs that target crucial host signaling or viral immunosuppressive proteins[334]. For example, administration of modified interferons, like Peginterferon (PEG-IFN) alfa-2a and 2b, have been developed to stimulate an innate immune response in combination with Ribavirin and tested in clinical trials with thousands of HCV patients[335]. Many Toll-like receptor agonists that activate the production of type I IFN are currently under development[336].

Likewise, host defense proteins/peptides endowed with either direct antiviral activity, immune regulation function, or both, are promising therapeutic drugs. Although the number of reported antiviral peptides is still very low, many natural and synthetic peptides have shown effectivity against diverse RNA viruses [337,338]. Among host defense proteins from nature, RNases are both involved in the host defense immunity system and display anti-infective activity [15,200]. The multifaceted properties of antimicrobial RNases, which combine among others, activation of the immune system, direct killing action, and the ability to inhibit the development of drug resistance encourage further research in this field[339]. Besides, novel methodologies such as the use of probiotics are developed to offer a cheaper affordable high-scale production and overcome the present main drawback of antimicrobial proteins pointed out by pharmaceutical companies[340]. In this review, we have described some representative RNases with demonstrated antiviral activity. We find references of RNases that can directly block viral replication by their ribonuclease activity or play a role in host PRR signaling pathways or cell stress responses. We also pointed out the advantages of host defense RNases in contrast to other drug candidates as effective antiviral agents. Notwithstanding, protein-based drugs are encountering difficulties in entering into the pharmaceutical market due to their poor bioavailability and high associated manufacturing costs. Currently, therapeutic proteins are well introduced in the treatment of chronic diseases in most developed countries, in contrast to infectious diseases that still remain non-attractive to investors. Hopefully, the present sanitary emergency will soon decant the balance toward a globally focused healthcare policy.

## Conclusions

Among the diversity of existing viruses, ssRNA viruses play an important role in threatening human health. Enveloped ssRNA viruses include not only HIV and HCV, which cause chronic infections and do not have effective vaccines, but also CoVs and RSV that induce acute respiratory symptoms and mostly threaten aged and infant population groups, respectively. There are many antiviral drugs available, but it is still challenging to design safe and effective antiviral drugs. One of the main difficulties encountered lies in the fact that viruses use host cells to replicate themselves. Therefore, nowadays all research efforts of the pharmaceutical industry are joint to seek new antiviral targets and new types of antiviral drugs. When infected by a virus, the host cells will activate many signaling pathways and secrete a variety of active factors to combat the foreign invader. Therefore, host-derived components such as RNases may be ideal candidates for the design of new antiviral drugs with unique properties. The host secretory proteins are nontoxic and can exert their action both within cells and in the extracellular space to prevent viral replication as well as regulate innate immunity. Although recent successes for HCV and HIV suggest that direct-acting antiviral small molecules remain the gold standard for antiviral drugs development, we cannot disregard the great potential of antimicrobial proteins and peptides with specific biological activities, such as RNases, as novel lead candidates to design antiviral drugs.
